# Cost-utility analysis of the UPRIGHT intervention promoting resilience in adolescents

**DOI:** 10.1186/s12888-023-04665-4

**Published:** 2023-03-17

**Authors:** Javier Mar, Igor Larrañaga, Oliver Ibarrondo, Ana González-Pinto, Carlota las Hayas, Ane Fullaondo, Irantzu Izco-Basurko, Jordi Alonso, Iñaki Zorrilla, Jessica Fernández-Sevillano, Esteban de Manuel, on behalf of the UPRIGHT Consortium, Nerea González, Maider Mateo-Abad, Patricia Pérez Martínez, Itziar Vergara, Silvia Gabrielli, Silvia Rizzi, Antoni Zwiefka, Dominik Krzyżanowski, Iwona Mazur, Luba Jakubowska, Renata Poteralska, Piotr Czyż, Urszula Andruszko, Paweł Błasiak, Katarzyna Krajewska, Grzegorz Pytlarz, Ilona Szczygieł-Grüdl, Odin Hjemdal, Roxanna Morote, Frederick Anyan, Dora Gudrun Gudmundsdottir, Solveig Karlsdottir, Hans Henrik Knoop, Mette Marie Ledertoug, Louise Tidmand, Anna Sigridur Olafsdottir, Unnur B. Arnfjord, Bryndis Jona Jonsdottir

**Affiliations:** 1 Osakidetza Basque Health Service, Debagoiena Integrated Health Organisation, Research Unit, Arrasate-Mondragón, Spain; 2 Biodonostia Health Research Institute, Donostia-San Sebastián, Spain; 3 Kronikgune Institute for Health Services Research, Barakaldo, Spain; 4 Osakidetza Basque Health Service, Araba University Hospital, Vitoria-Gasteiz, Spain; 5 University of the Basque Country (UPV/EHU), Vitoria-Gasteiz, Spain; 6 CIBER en Salud Mental (CIBERSAM), Madrid, Spain; 7 Bioaraba Health Research Institute, Vitoria-Gasteiz, Spain; 8 Health Services Research Group, IMIM- Institut Hospital del Mar d’Investigacions Mèdiques, Barcelona, Spain; 9 CIBER en Epidemiología Y Salud Pública (CIBERESP), Madrid, Spain; 10 Pompeu Fabra University (UPF), Barcelona, Spain; 11 University of Deusto, Department of Medicine, Bilbao, Spain

**Keywords:** Adolescents health, Resilience, Mental health, Mental disorders, Prevention, Simulation model, Health policy

## Abstract

**Background:**

As mental health in adulthood is related to mental status during adolescence, school-based interventions have been proposed to improve resilience. The objective of this study was to build a simulation model representing the natural history of mental disorders in childhood, adolescence and youth to estimate the cost-effectiveness of the UPRIGHT school-based intervention in promoting resilience and mental health in adolescence.

**Methods:**

We built a discrete event simulation model fed with real-world data (cumulative incidence disaggregated into eight clusters) from the Basque Health Service database (609,381 individuals) to calculate utilities (quality-adjusted life years [QALYs]) and costs for the general population in two scenarios (base case and intervention). The model translated changes in the wellbeing of adolescents into different risks of mental illnesses for a time horizon of 30 years.

**Results:**

The number of cases of anxiety was estimated to fall by 5,125 or 9,592 and those of depression by 1,269 and 2,165 if the effect of the intervention lasted 2 or 5 years respectively. From a healthcare system perspective, the intervention was cost-effective for all cases considered with incremental cost-utility ratios always lower than €10,000/QALY and dominant for some subgroups. The intervention was always dominant when including indirect and non-medical costs (societal perspective).

**Conclusions:**

Although the primary analysis of the trial did not did not detect significant differences, the UPRIGHT intervention promoting positive mental health was dominant in the economic evaluation from the societal perspective. Promoting resilience was more cost-effective in the most deprived group. Despite a lack of information about the spillover effect in some sectors, the economic evaluation framework developed principally for pharmacoeconomics can be applied to interventions to promote resilience in adolescents. As prevention of mental health disorders is even more necessary in the post-coronavirus disease-19 era, such evaluation is essential to assess whether investment in mental health promotion would be good value for money by avoiding costs for healthcare providers and other stakeholders.

**Supplementary Information:**

The online version contains supplementary material available at 10.1186/s12888-023-04665-4.

## Background

As mental health in adulthood is strongly dependent on mental status during childhood and adolescence [1], various school-based interventions have been proposed to promote complete mental health during youth [2, 3]. Among other aspects of mental health, these interventions address resilience, which is a general concept defined as the ability of an individual to adapt to life challenges or adversities while maintaining mental health and wellbeing [4, 5]. Although the literature contains numerous examples of school-based interventions aimed to boost adolescents’ resilience and wellbeing [6], they have rarely undergone economic evaluation [7]. Such evaluation is essential to assess whether investment in the promotion of mental health would be good value for money by avoiding costs for healthcare providers and other stakeholders associated with future mental disorders [8]. Should interventions be shown to be good value, this would indicate a need to overturn the current view that decision-makers do not need to prioritize mental health promotion [3]. Moreover, economic evaluations to date have aimed to measure the health benefits and costs of interventions versus the standard alternative only for the duration of trials [9, 10]. Within the INCLUSIVE trial, given the difficulty of assessing cost-utility, a cost-consequence analysis was performed without expressing the results as cost-effectiveness or cost-utility ratios [11]. Given this, the evaluation did not address the challenge of projecting the effectiveness of the intervention to medium- and long-term time horizons in terms of the prevention of mental disorders [12, 13]. Short-term assessments within the duration of trials explore improvements in positive mental health through scores on wellbeing scales [9, 10]. In contrast, the measurement of long-term outcomes implies investigating whether interventions succeed in preventing mental health problems. A conceptual model to bridge between short- and long-term focuses should be grounded on the dual-continua model of mental health taking into account the intertwined relationship between mental health and mental disease [14, 15]. Traditionally, mental health was reduced to the absence of mental illness [14]. Instead, the dual-continua model distinguishes between these concepts, representing them on different dimensions [14, 16].

The present study is framed within the UPRIGHT research project funded by the European Union’s Horizon 2020 programme (No. 754919) which developed a universal preventive resilience intervention to promote mental health among adolescents in schools [4]. It was designed as a whole school approach (for the school community including staff, adolescent students, and their families) to boost a culture of mental wellbeing by improving resilience and preventing mental disorders. To tackle the challenge of the long-term economic evaluation of this intervention, we have employed simulation modelling which is more routinely used in fields like pharmacoeconomics and cancer prevention.

The objective of this study was to build a simulation model representing the natural history of mental disorders in childhood, adolescence and youth to estimate the cost-effectiveness of the UPRIGHT intervention promoting resilience and mental health in adolescence.

## Methods

### Design

The study consisted of the comparison of two scenarios (usual care, implying delivery of the school curricula, and the UPRIGHT intervention including delivery of both the school curricula and the UPRIGHT intervention, a proactive program targeting all adolescents to improve their resilience) in terms of utility (quality-adjusted life years [QALYs]) and resource use (costs) by the mathematical representation of the natural history of mental disorders in adolescence and youth. For that purpose, we used information from the patient record system of the Basque Health Service (Spain), which is an anonymized well documented and maintained database of a whole population (real-world data). Based on these data, we built a discrete event simulation (DES) model [12] using the Arena® simulation tool.

DES is a specific technique that constructs a conceptual model which incorporates entities in a mathematical system and assigns them attributes or features. Entities represent individuals constituting a population to be followed along their pathway through the system generating the results required to understand the system and answer research questions [17, 18]. We chose to measure QALYs instead of disability-adjusted life years [19] or years lived with disability [20] because the cost per QALY or incremental cost-utility ratio (ICUR) is the format used to establish standard willingness-to-pay thresholds in the economic evaluation of interventions [21, 22]. The study took a societal perspective which implied identifying direct healthcare costs, direct non-medical costs and indirect costs and intervention costs. Impacts on mortality and health-related quality of life were also addressed. The cost-utility analysis included an annual discount of 3% for survival and costs [23]. The protocol of the study was approved by the Clinical Research Ethics Committee of the Basque Country (ref: PI2019078). UPRIGHT researchers, in collaboration with schools, obtained written informed consent from all participants, including teachers, adolescents and families (legal tutors also signing consent forms for adolescents’ participation).

### Conceptual model

The benefit of improving the resilience of adolescents is projected into adulthood in reductions in the risk of mental illness and increases in wellbeing [24]. The problem is that the literature does not provide data on the longitudinal evolution of this relationship in a way that it could be incorporated into the model. Recognizing this limitation, we projected the effect of the intervention only to the incidence of mental disorders for a time horizon of 30 years. Our rationale was that mental health is a state that can be assessed in each adolescent through questionnaires and that mental illnesses are events diagnosed following clinical criteria by general practitioners and psychiatrists during childhood, adolescence and youth [25]. Among other risk and protective factors, the development of mental disorders is partially related to mental wellbeing in early years as defined by effective coping skills, mindfulness, and resilience to external stressors [1, 4, 26].

**Fig. 1:**
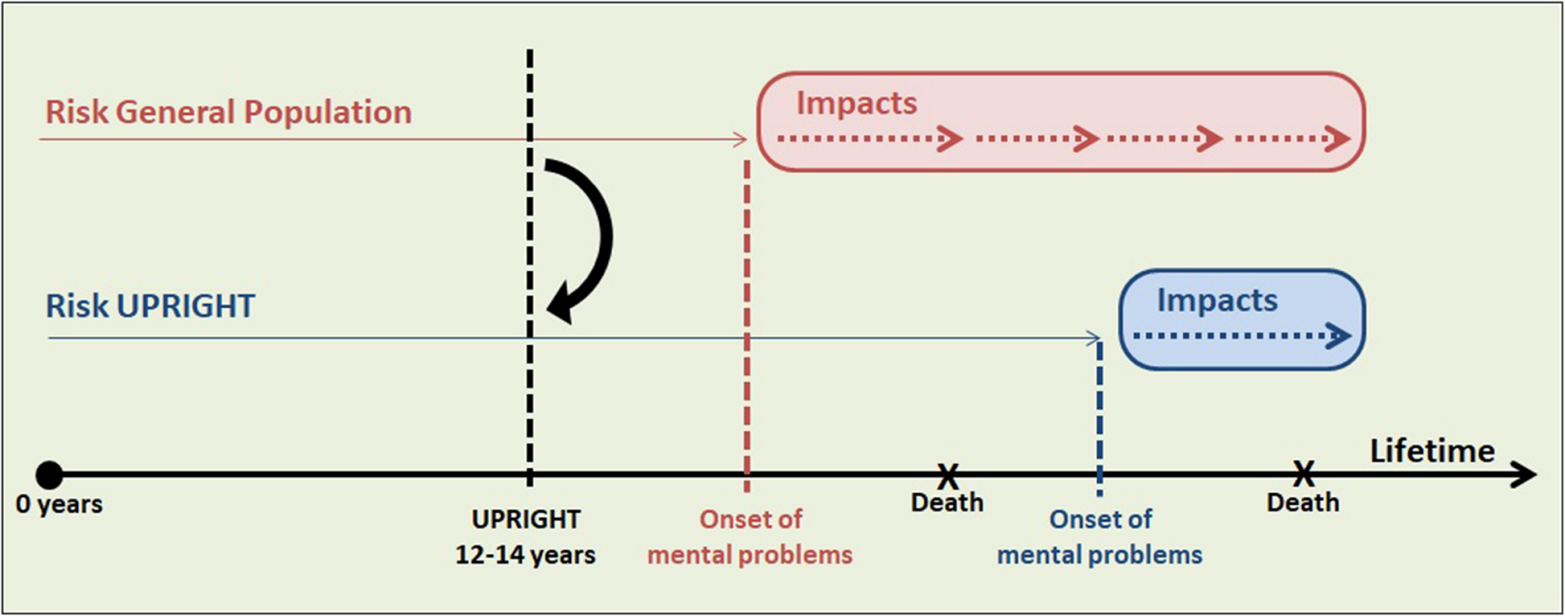
Theoretical representation of the natural history of mental disorders according to the level of psychological resilience in two scenarios of risk (general population and UPRIGHT population)

The natural history of mental disorders is graphically represented in Fig. 1. As resilience does not prevent all conditions [27], its protective effect was disaggregated by type of mental disorder. For this task, eight diagnostic clusters were defined: anxiety, attention deficit hyperactivity disorder (ADHD), conduct disorders, depression, substance use, psychosis and personality disorders, eating disorders, and self-harm. International Classification of Diseases (ICD)-9-Clinical Modification, ICD-10 and Anatomical, Therapeutic, Chemical classification system (ATC) codes were used to identify corresponding diagnoses (see Supplementary material, Table SM1**)**. Additionally, individuals who had any prescriptions for antidepressants (ATC N06A group) or antipsychotics (ATC N05A group) were included in the depression and psychosis categories respectively. Although the intervention was not expected to change the natural history of conditions in all these clusters, they were all included to represent the whole spectrum of mental disorders.

The study population was the 609,381 individuals who, as of December 31, 2018, were between 1 and 30 years old and were registered in the Basque Health Service (Table 1). At that time, the total Basque population was 2,180,449. All the data on demographic characteristics, mental disorders and healthcare resource use associated with mental disorders were extracted from the health service record system or dynamic population registry of the Basque Health Service’s institutional database, Oracle Business Intelligence (OBI) [28] which has been used to store clinical and administrative data since 2004 and is updated daily. A limitation of OBI is that it does not contain data from private practice records, and even though access to the health system is nearly universal for all residents, 20% of the population has double or complementary coverage through private insurance. This insurance does not, however, reimburse pharmacy prescriptions and that is why we also gathered data on prescriptions for antidepressants or antipsychotics, as mentioned above. Additional information needed to define simulation parameters was obtained from other official sources or the literature. We identified individuals with a diagnosis of mental health problems considering all the episodes of primary, emergency, outpatient and in-hospital care to measure the cumulative incidence of the eight diagnostic clusters.

**Table 1:** Univariate descriptive analysis of the sociodemographic characteristics of the study population

		General population		Population with mental disorders					*p*-value^a^
				No		Yes			
		N	%	N	%		N	%	
	Total	609,381		512,710			96,671		
Age, years	Mean	15.57		14.69			20.23		< 0.001
	1–10	182,731	30.0%	172,956	33.7%		9,775	10.1%	< 0.001
	11–20	210,817	34.6%	174,147	34.0%		36,670	37.9%	
	21–30	215,833	35.4%	165,607	32.3%		50,226	52.0%	
Gender	Female	296,556	48.7%	251,825	49.1%		44,731	46.3%	< 0.001
	Male	312,825	51.3%	260,885	50.9%		51,940	53.7%	
SES	Low	47,416	7.8%	36,945	7.2%		10,471	10.8%	< 0.001
	Medium-to-high	561,965	92.2%	475,765	92.8%		86,200	89.2%	

^a^Fisher’s exact test was used for categorical variables and Student’s t-test for continuous variables, *SES *Socioeconomic status

Statistical analyses for obtaining various parameters for the model were performed using Stata (version 14.0) or R (version 4.0.1). The cumulative incidence was calculated considering the date of onset of symptoms for each individual in the target population, for each cluster using the Aalen-Johansen estimator for competing risks (death and file closure due to individuals moving away) (function plotCIF from the R package Epi in CRAN) [29]. We also retrieved individuals’ socioeconomic status (SES) from their pharmacy co-payment category, which is based on household income [30], allowing cumulative incidence to be disaggregated by SES as well as gender. In this way, the model could also be employed for evaluating specific programs for reducing inequalities in high-risk populations. Nevertheless, in this study, we evaluated a universal intervention targeting the whole adolescent population.

### Intervention

The UPRIGHT universal preventive intervention to strengthen resilience was grounded on an individual, family and school staff framework (Figure SM1) and has been fully described elsewhere [4]. The economic evaluation sought to compare the epidemiological and economic impacts of the base case scenario with those of the alternative scenario of implementing the UPRIGHT intervention. The general population risk of mental disorders shaped the base case, and for the intervention scenario, the model captured the changes observed with specific questionnaires after deployment of the UPRIGHT intervention. Specifically, changes in questionnaire scores were translated to changes in disease incidence and the epidemiological and economic impacts could then be automatically calculated. Table SM2 shows the relationship between diagnostic clusters and scales used in UPRIGHT.

### General simulation model

The simulation model presented reproduces trajectories of 30 cohorts of the Basque general population up to 30 years old, one for each age group between 1 and 30 years of age (609,381 entities or individuals) according to the appearance of mental disorders across this range of ages and implementation of the UPRIGHT intervention. To relate SES to the risk of mental disorders in the model, the cumulative incidence of the eight clusters of mental disorder diagnoses considered was calculated separately for each sex-SES combination. The model started from 1 year of age to provide a complete representation of the natural history of mental disorders in childhood, adolescence and youth. Although the first 13 years have not been used in this work, it serves as a framework for possible studies analysing interventions that modify mental disorders that begin before the age of 13. Figure SM2 shows the model flow diagram, building on the conceptual model (Fig. 1). Table 2 summarizes the sources of the parameters.

**Table 2:** Sources of the model parameters

Parameters		Sources
Population characteristics Gender Age Socioeconomic status (SES) Cumulative incidence disaggregated by gender and Socioeconomic status Mortality	609,381 individuals Female (48.7%) (0–30 years) Low SES (7.8%) Empirical distribution Gompertz function	Basque Health Service database Table 1 Tables SM3-SM6 Table SM7
Utilities	By presence of mental disorders	Spanish National Health Survey (2012) [31, 32] Table SM8
Costs Direct healthcare costs Direct non-medical costs Indirect costs UPRIGHT Intervention costs	By cluster of mental disorder By cluster of mental disorder By cluster of mental disorder By student	Tables SM9-SM10 Basque Health Service database Literature [33] Literature [33] Microcosting
UPRIGHT Effectiveness Anxiety Depression Substance use Behavioral disorders	Questionnaires General Anxiety Disorder-7 Patient Health Questionnaire (PHQ*-*9) Items from WHO's Health Behavior in School-Aged Children (HBSC) survey	Table SM12 UPRIGHT trial
Systematic Review Effectiveness Anxiety Depression	Meta-analysis	Literature [27]

The development of the general simulation model was broken down into four sub-steps: (1) calculation of the SES- and gender-specific cumulative incidence rates for the diagnostic groups in the general population between 1 and 30 years of age to construct empirical distributions for time until the onset of mental disorders (Tables SM3-SM6), (2) retrieval of data on utilities and costs from available databases and literature, (3) estimation of the UPRIGHT intervention effectiveness comparing pre- and post-intervention data to modify times until events in a cloned population, and (4) construction and validation of the general simulation model combining all the information collected. Finally, both general and UPRIGHT populations (Fig. 1) were run from birth to 30 years to obtain outputs. As UPRIGHT was scheduled to be implemented when individuals were 12 years of age and it lasted 2 years, both costs and QALYs were recorded from 14 years old until the end of the time horizon (30 years).

**Fig. 2:**
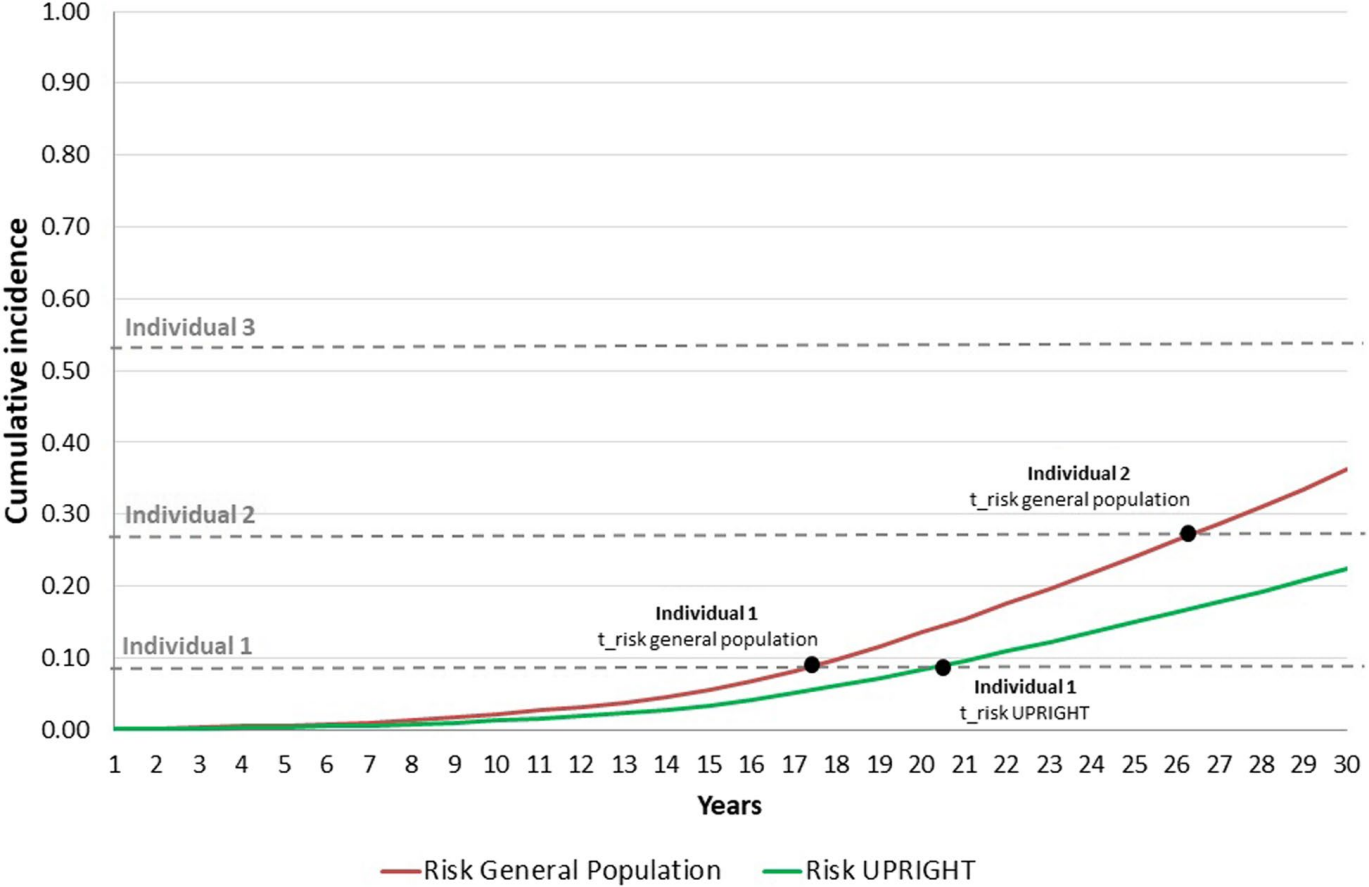
Three examples of assignment of time until the onset of mental disorders, based on the cumulative incidence (empirical distribution) and random numbers

When individuals were first entered into the model at age zero, they were assigned characteristics or personal attributes (gender and SES), and random numbers that made each individual’s life course different [34]. To guarantee that the two populations (general and UPRIGHT) had the same characteristics, once the attributes had been assigned, the population was cloned to produce two identical copies. In this way, the difference in the risk of mental disorders (as reflected in the cumulative incidences) due to the intervention was the only determinant of between-population differences in the trajectories of individuals. Second, the model calculated times until the onset of anxiety, ADHD, conduct disorders, depression, substance use, psychosis and personality disorders, eating disorders, and self-harm. Stochastic (or first-order) uncertainty was incorporated into the model by the aforementioned random numbers that acknowledged that individuals facing the same probabilities according to their attributes behave differently in the process of assigning events [34]. The main competing risks of the model were the onset of disease for each diagnostic cluster and death. It was assumed that after the onset of the mental disorders their consequences remained. Figure 2 illustrates the assignment of time until the onset of mental disorders, based on the empirical distribution of the cumulative incidence for the risk in each population until 30 years of age (the time horizon). In the examples, the intervention effect delays the onset of a mental disorder in individual 1, prevents the disorder in individual 2 and does not change the trajectory for individual 3. Finally, each individual was assigned costs and utilities according to the date of onset of the mental disorders. QALYs were obtained by multiplying the utilities by survival in years.

The time until death was obtained through a parametric survival analysis adjusted for independent variables (gender and presence of mental disorders) of the Basque population previously described using the Akaike Information Criterion [35]. Given the small number of deaths before 30 years old, mortality was not disaggregated by diagnostic cluster. After that, the Gompertz function was used to determine the time of death by gender and the presence of mental disorders (Table SM7). In this way, the reduction in mental disorder incidence rates due to the intervention also diminished the risk of death.

### Utilities

Utilities for individuals with and without mental disorders were extracted from a study [31] based on the 2012 Spanish National Health Survey (Spanish Ministry of Health, Social Services and Equality) [32], a nationwide survey conducted periodically in a representative sample of the non-institutionalized Spanish population aged ≥ 15 years. The 2012 survey included information from 21,007 individuals who responded to EuroQol's 5-level 5-dimension health status questionnaire, allowing the calculation of utility values for the Spanish general population as a function of various characteristics [36]. Table SM8 lists the utility values used for the general simulation model. We applied those values for each group according to gender, and socioeconomic status, from birth until 30 years old and differentiating between individuals with and without mental disorders.

### Direct healthcare costs

Direct healthcare costs were estimated by searching the aforementioned health service database for data on all resource use during 2018, including all contacts with nurses and general practitioners at healthcare centres, at home or by telephone in the case of primary care, and in the case of hospital care, all contacts with outpatient services and emergency services as well as hospitalizations. All the drugs prescribed to patients were also considered. Unit costs of healthcare resources for 2021 in euros (EUR, €) were taken from the Basque Health Service accounting system. Table SM9 lists the unit costs and Table SM10 the annual healthcare costs per patient updated to 2021.

### Direct non-medical costs and indirect costs

Direct non-medical and indirect costs were obtained from a systematic review, which provided specific estimates of the economic costs of brain disorders in Spain [37]. That study took a societal perspective and combined the methods and data retrieved from previous European-level research [33]. As ICD-10 codes were used to define the mental health categories, it was possible to relate them to the diagnostic groups defined for the UPRIGHT project in terms of cost per patient-year as direct non-medical and indirect costs. We did not consider costs due to premature mortality, intangible costs and costs of crime given a lack of data or appropriate assessment methods. Table SM10 presents the annual direct non-medical costs and indirect costs per patient that were updated to 2021 using the consumer price index.

### Intervention cost

The cost of the intervention per student was estimated by a microcosting approach. The Basque Education Department provided unit costs for teachers’ time and the staff training time unit cost was retrieved from a Basque Health Service list of official salaries. The time that each professional spent on intervention training and delivery was converted into training costs, implementation costs and material costs which were distributed among the mean number of students to obtain the total cost per student (€135.70). Table SM11 reports the disaggregated costs per student.

### Effectiveness of the intervention

The UPRIGHT intervention effect was estimated by comparing pre- and post-intervention scores on questionnaires administered during the cluster trial: the General Anxiety Disorder-7 [38] for anxiety, the Patient Health Questionnaire [39] for depression, and specific items of WHO's Health Behavior in School-Aged Children survey (HBSC) [40] for substance use and behavioral disorders (Table SM2). Given a lack of evidence about the duration of the intervention effect, two options were analyzed considering a waning effect after 2 and after 5 years.

The main analysis of the UPRIGHT intervention performed using mixed models did not detect significant differences between the two arms of the cluster trial in questionnaire scores indicating improvements in mental health associated with the intervention. As it was implemented from September 2018 to June 2021, the base case questionnaires were answered before the beginning of the coronavirus disease 2019 (COVID-19) pandemic and the intermediate and final ones during it. Given the added stress experienced by adolescents under conditions of exposure to COVID-19, lockdown and other measures to reduce social interaction, it did not seem appropriate to evaluate the intervention based on these findings. Therefore, the design of this study focused on developing the evaluation framework rather than measure the cost-utility of the UPRIGHT intervention. Nonetheless, seeking to test the capacity of the model to evaluate resilience interventions, the differences between intervention and control groups were analyzed by logistic regression considering only the individuals from the sample whose scores worsened from baseline to final measurements. Specifically, we found that the likelihood of worsening during the pandemic or changing from a low- to a high-risk group was significantly lower in the intervention group for anxiety (OR = 0.71; CIs: 0.50–1.00) and depression (OR = 0.66; CIs:0.47–0.91). On the other hand, UPRIGHT was not associated with similar changes in HBSC scores (Table SM12). These ORs were converted to relative risks (RRs) and multiplied by the base case incidence during the duration of the effect from the 14 years old of the intervention implementation (2 or 5 years) to estimate the cumulative incidence in the UPRIGHT arm adjusted for each cluster, gender and SES. Changing the incidence of one, two or five years also changes the cumulative incidence curve from that age up to 30 years. This property of empirical distributions is what we used to extend the time horizon up to 30 years even though the effect of the intervention wanes after one, two or five years.

As a sensitivity analysis, we ran the model incorporating the effectiveness of universal resilience-focused interventions targeting child and adolescent mental health in a school setting estimated in a systematic review by Dray et al. [27]. The meta-analysis showed significant effectiveness only for depression and anxiety and over a 1-year follow-up. Specifically, the cumulative incidence for the intervention arm was reduced with a standardized mean difference of -0.13 for depression and -0.18 for anxiety and only for 1 year [27].

### Validation

To validate the model, we used the AdViSHE tool, assessing four parts [41]: Part A: Validation of the conceptual model; Part B: Input data validation; Part C: Validation of the computerized model; and Part D: Operational validation. To test the external validity of the model outcomes, we assessed whether there were differences between the simulated age-specific cumulative incidences (from 1 to 30 years of age) and the observed ones. For that purpose, goodness-of-fit testing was conducted to obtain the correlation coefficient, normalised mean square error, fractional bias, fractional variance and the fraction of predictions within a factor of two.

### Medium-long term impacts

The model produced three types of results: (1) estimation of the medium-to-long term epidemiological and economic impacts of UPRIGHT, (2) cost-utility analysis to assess its cost-effectiveness from health system and societal perspectives, and (3) sensitivity analysis to test the robustness of the assumptions made about the effect size and duration.

## Results

### Validation

Part A: Validation of the conceptual model. Within the UPRIGHT Consortium, experts were asked to judge the appropriateness of the conceptual model. The expert panel, including psychiatrists, psychologists and mental health epidemiologists, reviewed the model and provided feedback helping to improve the model through an interactive process. Further, a cross-comparison was performed, comparing our approach with the conceptual models described by Le et al. in their systematic review [8] and this confirmed the comprehensiveness of our approach to representing the natural history of mental health in children, adolescents and young adults. Part B: Input data validation. The same experts judged the input parameters used to build the model to be appropriate. The agreement between our results and the cumulative incidence at 18 years measured by Dalsgaard [27] evidenced the goodness of fit of the model with external data on a key parameter. Other parameters, like mortality rates, utilities and costs, were taken from validated sources. Part C: Validation of the computerized model. To confirm that there were no substantive differences between the conceptual model and the DES model, an expert on model simulation not involved who had previously applied the AdViSHE tool to an obesity simulation model [42] reviewed the consistency of general and submodule architecture. Part D: Operational validation. All the goodness-of-fit statistics were within the established criteria when the simulated age-specific cumulative incidences were compared with the observed ones for each diagnostic cluster disaggregated by gender and SES (Table SM13).

### Epidemiological and economic impacts

As shown in Table 3 for the whole population included in the model (609,381 individuals), the number of cases of anxiety was estimated to fall by 5,125 if the intervention effect lasted 2 years, 9,592 if it lasted 5 years and 2,792 considering the meta-analysis result. Similarly, the number of cases of depression fell by 1,269, 2,165 and 442 respectively. In terms of mortality, the implementation of the intervention could be expected to avoid 14, 19 and 11 deaths respectively.

**Table 3:** Epidemiological impact of UPRIGHT on the incidence of mental disorders and number of deaths from birth to 30 years of age in the 30 cohorts (609,381 individuals) by diagnostic cluster as a function of the duration of the intervention’s effect

	Base case	UPRIGHT (2 years)		UPRIGHT (5 years)		Systematic Review [27]	
	Cases	Cases	Difference	Cases	Difference	Cases	Difference
Substance use	79,206	79,206	0	79,206	0	79.206	0
Anxiety	141,397	136,272	-5,125	131,805	-9,592	138.605	-2.792
Mood disorders	34,334	33,065	-1,269	32,169	-2,165	33.888	-446
Psychosis and personality disorders	19,988	19,988	0	19,988	0	19.988	0
Conduct disorders	41,309	41,309	0	41,309	0	41.309	0
ADHD	25,370	25,370	0	25,370	0	25.370	0
Eating disorders	9,785	9,785	0	9,785	0	9.785	0
Self-harm	2,614	2,614	0	2,614	0	2.614	0
Deaths	1,045	1,031	-14	1,026	-19	1.034	-11

*ADHD* Attention deficit hyperactivity disorder

Applying a discount of 3%, the intervention reduced the total costs by €69.16, €120.95 and €20.72 million depending on whether the effect lasted 2 years, 5 years or as in the meta-analysis (Table SM14). Considering only healthcare costs, the differences between the two scenarios were €50.01, €58.41 and €39.91 million respectively, the intervention costs being higher (€82.69 million). Table SM15 presents the same results but without discount.

### Cost-utility analysis

When the intervention cost-effectiveness was measured considering only healthcare costs (Table 4 with discount and SM16 without discount), the intervention was cost-effective in all cases with ICURs always lower than €10,000/QALY and dominant for some subgroups. In the discounted analysis the ICUR for the total population was €3,231/QALY for a 2-year effect, €1,939/QALY for a 5-year effect and €5,440/QALY with the effectiveness found in the systematic review. For all low-SES subgroups, the UPRIGHT scenario was dominant, i.e., it was associated with lower costs and higher QALYs than the usual care scenario. The pattern was the same for the undiscounted analyses, the intervention being dominant in more subgroups.

**Table 4:** Cost-utility analysis for healthcare costs and societal or total costs in euros with discount (3%) and disaggregated by gender and socioeconomic status by the duration of the intervention effect

**Discount (3%)**	Base case		UPRIGHT		∆ Cost	∆ QALY	ICUR
**Healthcare costs**	Cost (€)	QALY	Cost (€)	QALY			
**Total (2 years)**	4,059	10.81	4,112	10.82	53.64	0.02	3,231
Low SES male	5,430	10.72	5,421	10.73	-8.41	0.01	Dominant
High SES male	3,974	10.87	4,053	10.88	78.83	0.01	8,744
Low SES female	4,969	10.66	4,929	10.69	-39.80	0.02	Dominant
High SES female	3,951	10.77	3,994	10.79	42.58	0.02	1,815
**Total (5 years)**	4,059	10.81	4,098	10.83	39.84	0.02	1,939
Low SES male	5,430	10.72	5,386	10.74	-43.83	0.02	Dominant
High SES male	3,974	10.87	4,038	10.88	63.95	0.01	5,492
Low SES female	4,969	10.66	4,894	10.69	-74.64	0.03	Dominant
High SES female	3,951	10.77	3,984	10.79	33.30	0.03	1,176
**Total Systematic Review** [27]	4,059	10.81	4.129	10.82	70	0.01	5,440
Low SES male	5,430	10.72	5.470	10.73	40	0.01	5,178
High SES male	3,974	10.87	4.073	10.88	99	0.01	17,201
Low SES female	4,969	10.66	4.961	10.68	-8	0.02	Dominant
High SES female	3,951	10.77	4.003	10.79	52	0.02	2,651
**Discount (3%)**	Base case		UPRIGHT		∆ Cost	∆ QALY	ICUR
**Societal costs**	Cost (€)	QALY	Cost (€)	QALY			
**Total (2 years)**	12,757	10.81	12,643	10.82	-114	0.02	Dominant
Low SES male	28,256	10.72	27,933	10.73	-323	0.01	Dominant
High SES male	13,337	10.87	13,269	10.88	-68	0.01	Dominant
Low SES female	18,402	10.66	18,049	10.69	-353	0.02	Dominant
High SES female	10,467	10.77	10,347	10.79	-120	0.02	Dominant
**Total (5 years)**	12,757	10.81	12,558	10.83	-199	0.02	Dominant
Low SES male	28,256	10.72	27,798	10.74	-458	0.02	Dominant
High SES male	13,337	10.87	13,205	10.88	-132	0.01	Dominant
Low SES female	18,402	10.66	17,857	10.69	-545	0.03	Dominant
High SES female	10,467	10.77	10,255	10.79	-212	0.03	Dominant
**Total Systematic Review** [28]	12,757	10.81	12,722.80	10.82	-34.00	0.01	Dominant
Low SES male	28,256	10.72	28,103	10.73	-153	0.01	Dominant
High SES male	13,337	10.87	13,352	10.88	15	0.01	2,606
Low SES female	18,402	10.66	18,183	10.68	-219	0.02	Dominant
High SES female	10,467	10.77	10,412	10.79	-55	0.02	Dominant

*QALY* Quality-adjusted life year, *ICUR* Incremental cost-utility ratio, *SES* Socioeconomic status

When the cost-effectiveness of UPRIGHT was measured from the societal perspective including indirect and non-medical costs, the intervention was always dominant for discounted (Table SM17) and undiscounted (Table SM18) analyses. The saving generated for different stakeholders far outweighed the costs of the intervention.

## Discussion

The findings of this study show that the UPRIGHT psychoeducational intervention was associated with cost savings and health benefits when the societal costs were included, and therefore, it may be described as dominant. From the healthcare payer perspective, the intervention was cost-effective as the ICUR was always below the cost-effectiveness threshold defined in Spain [43]. Moreover, our research highlights that a framework traditionally applied in pharmacoeconomics [21] can also be employed for the economic evaluation of psychoeducational interventions to promote resilience in adolescents. In practical terms, it underlines that the prevention of mental disorders can be implemented as a sustainable public health policy based on an economic assessment. Moreover, as the cumulative incidence rates were disaggregated by gender and SES, the model could also be used to assess specific interventions targeting high-risk groups.

The use of economic evaluation to inform policy-makers about mental health promotion from the societal perspective implies examining the resource requirements and outcomes for different stakeholders [44]. Some of them, identified as non-healthcare sectors by the Second Panel recommendations for conducting cost-effectiveness analysis, are key for the assessment of mental illness costs like loss of productivity, social services, legal or criminal justice and education [42]. To complement the data on healthcare resource consumption retrieved from Basque real-world data, we used data on costs from the review by Parés-Badell et al., which defined direct non-medical costs as informal care, adaptation costs and transportation costs and indirect costs as those related to absence from work and early retirement [37]. Therefore, the scope of our approach based on considering only some stakeholders is a somewhat limited societal approach, as it did not include social services, legal or criminal justice, or education systems. The lack of information about the spillover effect that prevention of mental disorders actually has on social services, education or criminal justice represents a hurdle for a comprehensive economic evaluation of interventions promoting mental health. Despite this limitation, the UPRIGHT intervention gained health and saved costs [45].

The stress and measures to restrict social mobility associated with the COVID-19 pandemic hampered proper assessment of the effectiveness of the UPRIGHT intervention to strengthen resilience in the study (2020–2021) [46, 47]. Specifically, the repeated measures approach used to estimate the effectiveness was implemented in the context of adolescents experiencing added difficulties in their social lives and greater anxiety and stress. Nonetheless, in the case of mental wellbeing, while all participants showed deterioration from baseline, this was more marked in the control participants, and the magnitude of the reduction in mental wellbeing decreased over time for the intervention group. Therefore, our framework relies on the well-evidenced phenomenon that better coping with early adversity by improving resilience is an effective measure for preventing mental disorders [26]. Within the economic evaluation, we did not address the difficulties associated with the large-scale implementation of mental health promotion interventions beyond cost-effectiveness outcomes as highlighted by Le et al. in a systematic review [8]. They reported high dropout rates, indicating problems with acceptability, adherence, and feasibility regarding the interventions evaluated. Within the implementation of the UPRIGHT project, we identified some barriers such as a lack of commitment among school headteachers and problems with the participation of foster families. Teachers’ involvement was hampered by their job insecurity and work overload. Some saw the intervention as an extra strain on their working hours. These obstacles make it difficult to maintain the intervention over time. Its effectiveness might be enhanced if it were integrated into the school program in more year groups, but that would mean more pressure on schools.

We used real-world data to estimate the natural history of mental disorders by disaggregating them into clusters of diagnoses by gender and SES. The shaping of the model by multiple cohorts and clusters allowed us to obtain not only economic results but also epidemiological outcomes associated with the intervention in the 30 cohorts of the adolescent population at 13 years of age. The core of the model was the representation of the trajectories followed by individuals according to the risk of mental disorders adjusted for gender and SES. The cumulative incidence of mental disorders was calculated following the approach described by Dalsgaard et al., who estimated the same epidemiological indicators from a Danish registry [25]. The agreement between our results and the cumulative incidence at 18 years measured by Dalsgaard [25] is striking. The fact that two studies analysing different populations (Danish and Basque) found similar cumulative incidence rates of diagnosed mental disorders should strengthen confidence in real-world data as a source of parameters for modelling [48]. Studies from different countries also found similar incidence rates of dementia from registries based on electronic health records [28, 49, 50]. However, estimates of the prevalence of mental disorders collecting the presence of self-reported symptoms by adolescents through questionnaires resulted in higher figures than the cumulative incidence recorded in clinical databases [51–53]. These differences could be explained by the different methodology used, since surveys proactively ask for the presence of symptoms, while clinical databases include cases that contact the health system [54]. In our case, the public system only.

The main limitation of our study was the way the results of the UPRIGHT trial were incorporated into the model. The model would have been more robust if longitudinal data had been available relating mental health in terms of wellbeing and resilience in adolescence to the diagnosis of mental disorders in adulthood [28]. The questionnaires used measure the perception of adolescents of their interactions with the situations they are experiencing in family and school settings and other stressful events. It seems plausible that providing them with skills to cope with such events would improve their scores on these questionnaires. Nonetheless, they are surrogate outcomes that we have projected to the risk of mental disorders recorded as events in the health system. As underlined elsewhere, more long-term studies are needed to evidence this correlation between intermediate perceptions and final outcomes in terms of preventing mental disorders [27].

Resilience is a general concept defined as the ability of an individual to adapt to life challenges or adversities while maintaining mental health and wellbeing [4, 5]. Therefore, to move forward, we need to specify which clusters of mental disorders have lower incidence rates when resilience is improved. Our finding of anxiety and depression as the types of mental health problems that can be delayed or prevented by the intervention is consistent with the conclusion in a systematic review that universal resilience-focused interventions targeting adolescents are effective for depression and anxiety [27].

Our sensitivity analysis also suggested the intervention was cost-effective, finding an ICUR of €5,440/QALY. Another systematic review focused on school-based depression and anxiety prevention programs for young people also found small but significant effect sizes [55]. We overcame the lack of information about the waning effect by analysing different duration times and projecting them over the time horizon. Psychoeducational interventions require stable implementation in schools to maintain their effectiveness but the evaluation of such programs is beyond the scope of our research group’s activity.

## Conclusions

The UPRIGHT intervention promoting positive mental health was dominant from the societal perspective. Promoting resilience was more cost-effective in the most deprived group. Despite the lack of information about the spillover effect in some sectors, the economic evaluation framework developed principally for pharmacoeconomics can be applied to interventions to promote resilience in adolescents. The strength of the approach taken lies in the use of an epidemiological model built on a whole population registry to represent the intertwined natural history of mental health and mental illness. Given that economic models are still newcomers in mental health, more research is required to develop this approach to the same level as in other fields like cancer or cardiovascular disease. As prevention of mental health disorders is, if anything, even more necessary in the post-COVID-19 era, this type of evaluation is essential to assess whether investment in the promotion of mental health would be good value for money by avoiding costs for healthcare providers and other stakeholders.

## Supplementary Information

**Additional file 1.**

## Data Availability

The data that support the findings of this study are available from the Basque Health Service but restrictions apply to the availability of these data, which were used under license for the current study, and so are not publicly available. Data are however available from the authors upon reasonable request and with permission of the Basque Health Service.

## Abbreviations

QALYs: Quality-adjusted life years
DES: Discrete event simulation
ICUR: Incremental cost-utility ratio
ADHR: Attention deficit hyperactivity disorder
ICD: International Classification of Diseases
ATC: Anatomical, Therapeutic, Chemical classification system
SES: Socioeconomic status
HBSC: Health Behavior in School-Aged Children survey
