# Supplementary material for: Cost-utility analysis of the UPRIGHT intervention promoting resilience in adolescents
Source: BMC Psychiatry. 2023 Mar 17;23:178. doi: 10.1186/s12888-023-04665-4 (PMC10022565; doi:10.1186/s12888-023-04665-4)

Supplementary material. Development of a framework for the economic evaluation of interventions promoting resilience in adolescents.

Table SM1. International Classification of Diseases (ICD)-9-Clinical Modification, ICD-10 and Anatomical, Therapeutic, Chemical classification system (ATC) diagnostic codes used for each category of mental disorder.

In the identification process, the International Classification of Diseases (ICD)-9-Clinical Modification, ICD-10 and Anatomical, Therapeutic, Chemical classification system (ATC) provided the framework codes as shown in **Table SM1**, in the supplementary material (SM). In addition, we searched for individuals who had any prescriptions for antidepressants (ATC N06A group) or antipsychotics (ATC N05A group) to include them in the categories of depression and psychosis respectively. Although it was expected that the intervention would not change the natural history of psychosis and personality disorders, eating disorders, self-harm or ADHD, all of them were included in the study to represent the whole spectrum of mental disorders. We identified individuals with a diagnosis of mental health problems considering all the episodes of primary, emergency, outpatient and in-hospital care to measure the cumulative incidence of the eight diagnostic clusters.

	ICD-9	ICD-10	ATC
**Attention deficit hyperactivity disorder**	314.xx	F90.xx	
**Conduct disorders**	312.0x-312.2x, 312.4x-312.9x, 313.xx	F91.xx-F99.xx	
**Anxiety**	300.xx, 308.xx-309.xx	F40.xx-F49.xx	
**Depression**	296.xx-299.xx, 300.4, 311	F30.xx-F39.xx	Group ATC N06A
**Substance abuse**	303.xx-305.xx	F10.xx-F19.xx	
**Psychosis and personality disorders**	291.xx-292.xx, 295.xx<br>301.xx-302.xx, 312.3x	F20.xx-F29.xx<br>F60.xx-F69.xx	Group ATC N05A
**Eating disorders**	307.1x, 307.5x	F50.xx	
**Self-harm**	V62.84, E95x.xx	R45.851, T14.91, X71.xx-X83.xx	

Table SM2. Relationship between diagnostic clusters and scales used in UPRIGHT.

Measurement (Scale)	ADHD	Conduct disorders	Anxiety	Depression	Substance use	Psychosis	Eating disorders	Self-harm
GAD-7								
PHQ-9								
HBSC<br>Drugs use								
HBSC<br>Fighting and bullying								

GAD-7: General Anxiety Disorder-7; PHQ-9: Patient Health Questionnaire; HBSC: WHO's Health Behavior in School-Aged Children

Table SM3. Cumulative incidence (%) by type of mental disorder from 1 to 30 years of age for male population with low socioeconomic status.

Age	ADHD	Conduct disorders	Anxiety	Depression	Substance use	Psychosis and personality disorders	Eating disorders	Self-harm
**1**	0.01%	0.12%	0.01%	0.03%	0.00%	0.01%	0.06%	0.00%
**2**	0.03%	0.38%	0.06%	0.20%	0.00%	0.06%	0.14%	0.00%
**3**	0.12%	0.91%	0.15%	0.37%	0.00%	0.10%	0.17%	0.00%
**4**	0.26%	1.50%	0.26%	0.51%	0.00%	0.14%	0.24%	0.01%
**5**	0.49%	2.21%	0.37%	0.61%	0.00%	0.21%	0.28%	0.01%
**6**	1.06%	3.09%	0.53%	0.73%	0.00%	0.27%	0.34%	0.01%
**7**	1.92%	4.06%	0.81%	0.82%	0.00%	0.33%	0.38%	0.01%
**8**	2.83%	5.15%	1.17%	0.91%	0.00%	0.41%	0.41%	0.01%
**9**	3.78%	6.11%	1.50%	0.99%	0.00%	0.54%	0.45%	0.01%
**10**	4.59%	7.21%	1.97%	1.09%	0.01%	0.68%	0.47%	0.01%
**11**	5.27%	8.14%	2.60%	1.21%	0.01%	0.79%	0.48%	0.03%
**12**	5.80%	8.97%	3.19%	1.32%	0.04%	0.93%	0.52%	0.04%
**13**	6.55%	9.96%	3.72%	1.42%	0.14%	1.11%	0.55%	0.06%
**14**	7.19%	10.82%	4.41%	1.56%	0.42%	1.36%	0.60%	0.09%
**15**	7.51%	11.26%	5.19%	1.80%	1.00%	1.63%	0.65%	0.12%
**16**	7.67%	11.61%	6.03%	2.12%	1.78%	2.03%	0.71%	0.14%
**17**	7.84%	12.08%	7.05%	2.48%	2.86%	2.61%	0.84%	0.17%
**18**	7.96%	12.39%	8.42%	3.03%	4.16%	3.32%	0.86%	0.20%
**19**	8.11%	12.66%	9.57%	3.74%	5.70%	4.15%	0.87%	0.30%
**20**	8.16%	13.02%	11.03%	4.37%	7.33%	4.94%	0.89%	0.33%
**21**	8.27%	13.43%	12.50%	5.18%	9.08%	5.64%	0.91%	0.43%
**22**	8.39%	13.79%	14.20%	6.03%	11.04%	6.56%	0.96%	0.48%
**23**	8.45%	14.10%	15.77%	6.99%	12.55%	7.54%	0.99%	0.57%
**24**	8.50%	14.72%	17.75%	8.31%	14.48%	8.98%	1.07%	0.77%
**25**	8.60%	15.08%	19.63%	9.51%	16.84%	10.30%	1.07%	0.88%
**26**	8.85%	15.58%	21.37%	10.63%	19.21%	11.85%	1.07%	1.00%
**27**	9.08%	16.11%	23.18%	12.13%	21.08%	13.50%	1.07%	1.15%
**28**	9.08%	16.78%	25.01%	13.00%	22.92%	15.05%	1.07%	1.54%
**29**	9.08%	17.05%	26.75%	14.34%	24.79%	16.12%	1.07%	1.67%
**30**	9.08%	17.05%	28.82%	15.64%	26.34%	18.71%	1.33%	2.19%

Table SM4. Cumulative incidence (%) by type of mental disorder from 1 to 30 years of age for male population with medium-high socioeconomic status.

Age	ADHD	Conduct disorders	Anxiety	Depression	Substance use	Psychosis and personality disorders	Eating disorders	Self-harm
**1**	0.01%	0.07%	0.02%	0.02%	0.00%	0.01%	0.03%	0.00%
**2**	0.03%	0.27%	0.05%	0.09%	0.00%	0.03%	0.09%	0.00%
**3**	0.07%	0.54%	0.09%	0.18%	0.00%	0.05%	0.14%	0.00%
**4**	0.15%	0.91%	0.14%	0.24%	0.00%	0.08%	0.17%	0.00%
**5**	0.30%	1.30%	0.20%	0.29%	0.00%	0.11%	0.20%	0.00%
**6**	0.64%	1.77%	0.32%	0.34%	0.00%	0.13%	0.22%	0.00%
**7**	1.21%	2.31%	0.44%	0.38%	0.00%	0.16%	0.24%	0.00%
**8**	1.80%	2.84%	0.59%	0.42%	0.00%	0.20%	0.26%	0.00%
**9**	2.45%	3.39%	0.80%	0.47%	0.00%	0.25%	0.29%	0.00%
**10**	2.95%	3.91%	1.06%	0.51%	0.00%	0.28%	0.31%	0.00%
**11**	3.39%	4.39%	1.33%	0.55%	0.00%	0.33%	0.33%	0.00%
**12**	3.79%	4.81%	1.61%	0.60%	0.01%	0.38%	0.35%	0.01%
**13**	4.27%	5.27%	1.90%	0.64%	0.04%	0.44%	0.37%	0.01%
**14**	4.72%	5.70%	2.20%	0.71%	0.15%	0.51%	0.39%	0.02%
**15**	5.01%	5.96%	2.54%	0.78%	0.40%	0.60%	0.43%	0.02%
**16**	5.22%	6.19%	2.95%	0.86%	0.84%	0.70%	0.46%	0.03%
**17**	5.38%	6.38%	3.48%	0.97%	1.39%	0.82%	0.49%	0.04%
**18**	5.49%	6.55%	4.09%	1.09%	2.05%	0.94%	0.51%	0.04%
**19**	5.56%	6.65%	4.81%	1.23%	2.81%	1.10%	0.53%	0.06%
**20**	5.61%	6.75%	5.64%	1.40%	3.64%	1.26%	0.55%	0.07%
**21**	5.64%	6.84%	6.51%	1.59%	4.52%	1.42%	0.57%	0.08%
**22**	5.68%	6.91%	7.48%	1.81%	5.50%	1.58%	0.58%	0.10%
**23**	5.71%	6.97%	8.44%	2.02%	6.48%	1.75%	0.60%	0.11%
**24**	5.76%	7.06%	9.51%	2.24%	7.52%	1.92%	0.61%	0.12%
**25**	5.79%	7.15%	10.58%	2.56%	8.63%	2.09%	0.63%	0.15%
**26**	5.81%	7.22%	11.74%	2.84%	9.70%	2.26%	0.64%	0.16%
**27**	5.85%	7.30%	12.94%	3.16%	10.80%	2.47%	0.64%	0.17%
**28**	5.86%	7.35%	14.11%	3.50%	11.85%	2.66%	0.65%	0.19%
**29**	5.88%	7.40%	15.33%	3.87%	12.82%	2.89%	0.65%	0.21%
**30**	5.88%	7.50%	16.68%	4.36%	13.86%	3.18%	0.66%	0.23%

Table SM5. Cumulative incidence (%) by type of mental disorder from 1 to 30 years of age for female population with low socioeconomic status.

Age	ADHD	Conduct disorders	Anxiety	Depression	Substance use	Psychosis and personality disorders	Eating disorders	Self-harm
**1**	0.01%	0.08%	0.02%	0.04%	0.00%	0.03%	0.04%	0.00%
**2**	0.02%	0.22%	0.05%	0.08%	0.00%	0.06%	0.08%	0.00%
**3**	0.03%	0.50%	0.08%	0.11%	0.00%	0.07%	0.14%	0.00%
**4**	0.08%	0.80%	0.18%	0.12%	0.00%	0.08%	0.20%	0.00%
**5**	0.12%	1.14%	0.26%	0.14%	0.00%	0.09%	0.23%	0.00%
**6**	0.28%	1.56%	0.40%	0.18%	0.00%	0.13%	0.26%	0.00%
**7**	0.65%	2.06%	0.60%	0.20%	0.00%	0.15%	0.28%	0.00%
**8**	1.01%	2.64%	0.92%	0.21%	0.01%	0.16%	0.34%	0.00%
**9**	1.46%	3.20%	1.27%	0.25%	0.01%	0.20%	0.37%	0.00%
**10**	1.72%	3.82%	1.77%	0.26%	0.02%	0.23%	0.43%	0.00%
**11**	1.87%	4.40%	2.27%	0.30%	0.02%	0.27%	0.49%	0.00%
**12**	2.05%	4.88%	2.71%	0.32%	0.04%	0.31%	0.59%	0.00%
**13**	2.29%	5.64%	3.54%	0.40%	0.09%	0.38%	0.71%	0.01%
**14**	2.54%	6.36%	4.48%	0.62%	0.34%	0.50%	0.92%	0.05%
**15**	2.69%	6.81%	5.84%	0.81%	0.88%	0.74%	1.14%	0.11%
**16**	2.80%	7.15%	7.58%	1.22%	1.48%	0.94%	1.43%	0.15%
**17**	2.91%	7.49%	9.74%	1.53%	2.38%	1.21%	1.66%	0.21%
**18**	2.97%	7.86%	11.89%	1.95%	3.39%	1.55%	1.89%	0.28%
**19**	3.06%	8.10%	14.12%	2.50%	4.33%	1.94%	1.99%	0.33%
**20**	3.09%	8.27%	16.41%	2.88%	5.18%	2.20%	2.10%	0.39%
**21**	3.11%	8.47%	19.07%	3.39%	6.42%	2.51%	2.20%	0.44%
**22**	3.15%	8.65%	21.90%	4.16%	7.59%	2.95%	2.40%	0.55%
**23**	3.17%	8.92%	24.79%	4.75%	8.89%	3.34%	2.47%	0.60%
**24**	3.21%	9.14%	27.47%	5.37%	10.26%	4.00%	2.63%	0.82%
**25**	3.24%	9.33%	30.49%	6.37%	12.02%	4.46%	2.70%	0.90%
**26**	3.24%	9.51%	32.90%	7.26%	13.18%	4.68%	2.79%	0.94%
**27**	3.24%	9.82%	34.80%	7.95%	14.82%	5.31%	2.85%	1.00%
**28**	3.24%	9.89%	37.60%	8.95%	15.82%	5.85%	3.05%	1.33%
**29**	3.24%	10.08%	40.07%	10.56%	17.24%	6.23%	3.14%	1.42%
**30**	3.24%	10.26%	42.58%	12.17%	17.96%	6.41%	3.14%	1.42%

Table SM6. Cumulative incidence (%) by type of mental disorder from 1 to 30 years of age for female population with medium-to-high socioeconomic status.

Age	ADHD	Conduct disorders	Anxiety	Depression	Substance use	Psychosis and personality disorders	Eating disorders	Self-harm
**1**	0.01%	0.08%	0.01%	0.01%	0.00%	0.01%	0.04%	0.00%
**2**	0.01%	0.20%	0.04%	0.03%	0.00%	0.02%	0.09%	0.00%
**3**	0.03%	0.37%	0.07%	0.05%	0.00%	0.03%	0.12%	0.00%
**4**	0.05%	0.59%	0.11%	0.07%	0.00%	0.03%	0.15%	0.00%
**5**	0.09%	0.80%	0.16%	0.08%	0.00%	0.04%	0.18%	0.00%
**6**	0.19%	1.05%	0.24%	0.10%	0.00%	0.05%	0.22%	0.00%
**7**	0.40%	1.33%	0.36%	0.11%	0.00%	0.07%	0.24%	0.01%
**8**	0.63%	1.65%	0.51%	0.12%	0.00%	0.08%	0.26%	0.01%
**9**	0.89%	1.99%	0.72%	0.13%	0.00%	0.10%	0.29%	0.01%
**10**	1.10%	2.31%	1.01%	0.15%	0.00%	0.12%	0.31%	0.01%
**11**	1.27%	2.58%	1.28%	0.17%	0.01%	0.13%	0.34%	0.01%
**12**	1.41%	2.84%	1.59%	0.19%	0.01%	0.16%	0.39%	0.01%
**13**	1.56%	3.16%	1.94%	0.22%	0.04%	0.19%	0.47%	0.02%
**14**	1.70%	3.46%	2.42%	0.29%	0.18%	0.24%	0.62%	0.04%
**15**	1.80%	3.64%	3.10%	0.38%	0.41%	0.30%	0.82%	0.07%
**16**	1.88%	3.82%	4.00%	0.49%	0.73%	0.39%	1.03%	0.09%
**17**	1.94%	3.96%	5.14%	0.67%	1.16%	0.49%	1.22%	0.12%
**18**	2.00%	4.09%	6.41%	0.84%	1.60%	0.60%	1.38%	0.15%
**19**	2.04%	4.19%	7.78%	1.01%	2.19%	0.68%	1.53%	0.17%
**20**	2.06%	4.28%	9.29%	1.19%	2.81%	0.76%	1.65%	0.18%
**21**	2.09%	4.35%	10.84%	1.42%	3.43%	0.85%	1.75%	0.20%
**22**	2.12%	4.41%	12.48%	1.63%	4.12%	0.92%	1.85%	0.22%
**23**	2.13%	4.48%	14.10%	1.93%	4.90%	1.02%	1.93%	0.24%
**24**	2.16%	4.53%	15.79%	2.21%	5.65%	1.10%	2.00%	0.25%
**25**	2.18%	4.60%	17.54%	2.55%	6.54%	1.19%	2.07%	0.28%
**26**	2.20%	4.68%	19.28%	2.94%	7.41%	1.30%	2.12%	0.29%
**27**	2.20%	4.76%	21.04%	3.30%	8.24%	1.40%	2.17%	0.31%
**28**	2.20%	4.79%	22.96%	3.87%	9.03%	1.54%	2.21%	0.35%
**29**	2.21%	4.85%	24.88%	4.49%	9.83%	1.69%	2.28%	0.37%
**30**	2.21%	4.93%	27.13%	5.45%	10.66%	1.90%	2.37%	0.42%

Table SM7. Distribution and parameters for the time until event function for death.

		Death
**Type of function**		Gompertz
Sex (x1)	Women	0.000
	Men	0.638
Mental health problem (x2)	No	0.000
	Yes	0.431
Constant (x0)		-30.261
Beta		0.001

As can be seen in **Table SM7**, a Gompertz distribution was selected to model time to death and this was expressed with the formula below:

$$Time\ to\ event\ (Gompertz) = \frac{1}{\beta} * ln(1 - \frac{\beta}{\alpha} * ln(1-u) * e^{-\beta})$$

where:

$$ln(\alpha) = x_0 + x_1 * sex + x_2 * presence\ of\ mental\ health\ problem.$$

The equations included a uniformly distributed random factor between 0 and 1 (u) and two parameters α and β that defined the characteristics of the distribution.

Table SM8. Utilities for individuals with and without mental health problems extracted from the 2012 Spanish National Health Survey (EQ-5D-5L).

	Mental health problems	
	No	Yes
Male with low SES	0.9851	0.9082
Male with medium-to-high SES	0.9875	0.8942
Female with low SES	0.9856	0.8807
Female with medium-to-high SES	0.9880	0.8448

Table SM9. Unit costs of the healthcare resources obtained from the Basque Health Service for 2021.

Resource	Euros (€)
PC nurse (centre)	12.31
PC nurse (telephone)	6.16
PC nurse (home)	22.37
General practitioner (centre)	27.91
General practitioner (telephone)	13.95
General practitioner (home)	39.09
Outpatient services (first)	136.55
Outpatient services (second)	80.31
Emergency services	172.68
Hospitalisation (per day)	486.02
Home hospitalisation (per day)	349.15

PC: primary care

Table SM10. Direct healthcare cost, direct non-medical cost and indirect cost per person (in €) disaggregated by diagnostic group.

	Healthy population	Substance abuse	Anxiety	Mood disorders	Psychosis	ADHD	Conduct disorders	Eating disorders	Self-harm	2 or more diagnoses
**Primary care**										
1-12 years	19.962	45.730	16.135	16.520	15.420	15.494	16.542	17.958	12.075	16.368
13-18 years	43.881	91.536	90.771	69.181	68.629	56.736	64.215	69.654	101.095	106.122
19-24 years	57.394	87.708	118.008	99.408	88.174	69.344	78.358	88.239	91.986	132.134
25-30 years	58.664	87.319	122.432	116.015	83.109	66.230	81.479	83.486	96.833	146.441
**Hospital care cost**										
1-12 years	249.532	188.736	382.793	609.057	850.128	411.899	407.668	448.813	400.664	834.797
13-18 years	169.918	340.340	369.488	495.961	477.167	303.361	313.406	606.681	603.420	1,340.595
19-24 years	161.619	340.697	330.237	434.156	568.007	191.287	221.019	350.702	881.225	1,046.204
25-30 years	195.941	354.709	378.249	554.961	366.830	167.253	249.729	506.568	531.169	1,144.294
**Drug prescription cost**										
1-12 years	33.204	550.634	105.303	119.232	107.750	112.576	106.262	103.288	79.347	106.673
13-18 years	29.323	133.303	133.925	148.710	115.868	114.098	122.174	120.835	73.693	141.839
19-24 years	35.846	108.959	114.428	124.946	143.132	113.196	113.513	118.332	89.123	113.774
25-30 years	37.466	132.972	109.382	76.933	115.491	118.041	105.612	131.027	40.397	103.459
**Direct non-medical cost**	0.000	982.311	27.192	531.377	660.539	3,422.793	3,422.793	50.985	660.539	-
**Indirect cost**	0.000	1,489.895	1,074.084	1,813.933	13,261.765	0.000	0.000	101.970	1,813.933	-

Table SM11. Cost per student receiving the two years of the UPRIGHT intervention.

Cost component	Cost (€)
Training cost	80.81
Implementation cost	44.92
Material cost	10.00
Total cost	135.73

Table SM12. Multivariate statistical analysis estimating the likelihood of worsening because of the pandemia or changing from a low- to a high-risk group expressed as an odd ratio (OR) and confidence intervals.

	GAD-7[a]	PHQ-9[a]	HBSC - Drug use[a]	HBSC - Fighting and bullying[a]
**Control group**	Reference	Reference	Reference	Reference
**Intervention group**	0.71 (0.50-1.00)*	0.66 (0.47-0.91)*	0.90 (0.60-1.34)	1.11 (0.89-1.38)

* p-value ≤ 0,05, ** p-value ≤ 0,01.

[a] Calculated using logistic binary regression.

Table SM13. Model validation through comparison of the observed and simulated cumulative incidences. Goodness-of-fit test for each diagnostic group stratified by sex and SES. To validate the model, the correlation coefficient and the FAC2 were required to be higher than 0.8, the normalised mean squared error lower than 0.5, and the fractional bias and fractional variance between -0.5 and 0.5.

Low SES male	ADHD	Conduct disorders	Anxiety	Mood disorders	Substance use	Psychosis and personality disorders	Eating disorders	Self-harm
**R: > 0.8**	0.966	0.967	0.967	0.967	0.967	0.967	0.965	0.966
**NMSE: < 0.5**	0.000	0.000	0.000	0.002	0.000	0.001	0.005	0.016
**FB: [-0.5, 0.5]**	0.016	-0.009	-0.003	-0.038	-0.007	-0.016	-0.055	-0.076
**FV: [-0.5, 0.5]**	0.021	0.022	0.012	0.022	0.016	0.018	0.094	0.073
**FAC2: > 0.8**	1.000	1.000	1.000	1.000	1.000	1.000	1.000	1.000
**Medium-to-High SES male**	**ADHD**	**Conduct disorders**	**Anxiety**	**Mood disorders**	**Substance use**	**Psychosis and personality disorders**	**Eating disorders**	**Self-harm**
**R: > 0.8**	0.967	0.967	0.967	0.967	0.967	0.967	0.967	0.966
**NMSE: < 0.5**	0.000	0.000	0.000	0.000	0.000	0.001	0.001	0.012
**FB: [-0.5, 0.5]**	0.005	0.002	0.011	0.008	-0.009	-0.015	0.034	-0.070
**FV: [-0.5, 0.5]**	0.008	0.004	0.011	0.016	0.007	0.021	0.017	0.072
**FAC2: > 0.8**	1.000	1.000	1.000	1.000	0.917	1.000	1.000	1.000
**Low SES female**	**ADHD**	**Conduct disorders**	**Anxiety**	**Mood disorders**	**Substance use**	**Psychosis and personality disorders**	**Eating disorders**	**Self-harm**
**R: > 0.8**	0.967	0.967	0.967	0.967	0.967	0.966	0.966	0.966
**NMSE: < 0.5**	0.000	0.000	0.000	0.003	0.001	0.001	0.002	0.009
**FB: [-0.5, 0.5]**	0.001	0.019	-0.005	-0.041	-0.014	0.014	0.037	0.067
**FV: [-0.5, 0.5]**	0.019	0.014	0.005	0.031	0.017	0.024	0.043	0.054
**FAC2: > 0.8**	0.833	1.000	1.000	0.917	1.000	1.000	1.000	1.000
**Medium-to-High SES female**	**ADHD**	**Conduct disorders**	**Anxiety**	**Mood disorders**	**Substance use**	**Psychosis and personality disorders**	**Eating disorders**	**Self-harm**
**R: > 0.8**	0.967	0.967	0.967	0.967	0.967	0.967	0.967	0.967
**NMSE: < 0.5**	0.000	0.000	0.000	0.000	0.000	0.000	0.000	0.001
**FB: [-0.5, 0.5]**	-0.018	0.009	0.004	0.010	0.001	-0.001	0.009	-0.019
**FV: [-0.5, 0.5]**	0.016	0.006	0.003	0.007	0.004	0.019	0.007	0.017
**FAC2: > 0.8**	1.000	1.000	1.000	1.000	1.000	1.000	1.000	1.000

R: correlation coefficient; NMSE: normalised mean square error; FB: fractional bias; FV: fractional variance; FAC2: fraction of predictions within a factor of two

Table SM14. Cost savings expressed in millions of euros achieved by the UPRIGHT intervention in the total population (30 cohorts) from 14 to 30 years old for different cost components with a discount (3%) and according to the intervention duration.

	Base case	UPRIGHT (2 years)		UPRIGHT (5 years)		Systematic Review[28]	
	Costs	Costs	Difference	Costs	Difference	Costs	Difference
Direct healthcare costs	2,473.28	2,423.27	-50.01	2,414.86	-58.41	2,433.36	-39.91
Primary care	436.00	431.24	-4.75	429.45	-6.55	432.67	-3.33
Hospital care	1,665.21	1,626.84	-38.37	1,621.89	-43.33	1,633.75	-31.47
Drug prescription	372.07	365.18	-6.88	363.53	-8.54	366.95	-5.12
Direct non-medical costs	2,646.66	2,634.26	-12.39	2,632.55	-14.11	2,637.49	-9.17
Indirect costs	2,653.90	2,564.45	-89.45	2,522.78	-131.12	2,599.58	-54.33
Intervention costs	0.00	82.69	82.69	82.69	82.69	82.69	82.69
Total costs	7,773.84	7,704.68	-69.16	7,652.88	-120.95	7,753.12	-20.72

Table SM15. Total costs for different cost categories without a discount expressed in millions of euros (M€).

	Base case	UPRIGHT (2 years)		UPRIGHT (5 years)		Systematic Review[38]	
	**Cost (M€)**	**Cost (M€)**	**Difference (M€)**	**Cost (M€)**	**Difference (M€)**	**Cost (M€)**	**Difference (M€)**
**Direct healthcare costs**	3,901.02	3,819.80	-81.22	3,805.70	-95.32	3,835.57	-65.45
Primary care costs	687.92	680.25	-7.68	677.23	-10.70	682.52	-5.41
Hospital care costs	2,628.33	2,565.14	-63.19	2,556.63	-71.70	2,576.11	-52.22
Drug prescription costs	584.77	574.42	-10.35	571.84	-12.93	576.94	-7.83
**Direct non-medical costs**	4,166.92	4,146.70	-20.22	4,143.90	-23.03	4,151.66	-15.26
**Indirect costs**	4,610.40	4,471.81	-138.58	4,404.26	-206.14	4,525.38	-85.01
**Intervention costs**	0.00	82.69	82.69	82.69	82.69	82.69	82.69
**Total costs**	12,678.34	12,521.01	-157.33	12,436.54	-241.80	12,595.30	-83.04

Table SM16. Cost-utility analysis for healthcare in costs (euros) without discount and disaggregated by sex and socioeconomic status.

	**Base case**		**UPRIGHT (2 years)**		**Δ Cost**	**Δ QALY**	**ICUR**
	**Cost (€)**	**QALY**	**Cost (€)**	**QALY**			
**Total**	6,401.54	16.24	6,403.96	16.27	2.42	0.03	92.58
**Low SES male**	8,736.65	16.12	8,640.11	16.14	-96.54	0.02	Dominant
**High SES male**	6,230.93	16.36	6,276.57	16.37	45.64	0.01	3,283.30
**Low SES female**	7,984.05	16.00	7,831.22	16.04	-152.83	0.04	Dominant
**High SES female**	6,240.81	16.17	6,223.20	16.21	-17.60	0.04	Dominant
	**Base case**		**UPRIGHT (5 years)**		**Δ Cost**	**Δ QALY**	**ICUR**
	**Cost (€)**	**QALY**	**Cost (€)**	**QALY**			
**Total**	6,401.54	16.24	6,380.82	16.28	-20.73	0.03	Dominant
**Low SES male**	8,736.65	16.12	8,581.50	16.14	-155.14	0.02	Dominant
**High SES male**	6,230.93	16.36	6,251.89	16.37	20.96	0.02	1,154.14
**Low SES female**	7,984.05	16.00	7,772.62	16.06	-211.43	0.05	Dominant
**High SES female**	6,240.81	16.17	6,207.33	16.21	-33.48	0.05	Dominant
	**Base case**		**Systematic Review[38]**		**Δ Cost**	**Δ QALY**	**ICUR**
	**Cost (€)**	**QALY**	**Cost (€)**	**QALY**			
**Total**	6,401.54	16.24	6,429.84	16.27	28.29	0.02	1,380.84
**Low SES male**	8,736.65	16.12	8,716.10	16.13	-20.55	0.01	Dominant
**High SES male**	6,230.93	16.36	6,307.59	16.37	76.66	0.01	8,617.94
**Low SES female**	7,984.05	16.00	7,881.32	16.03	-102.74	0.03	Dominant
**High SES female**	6,240.81	16.17	6,238.09	16.20	-2.71	0.03	Dominant

QALY: quality adjusted life year; ICUR: incremental cost-utility ratio.

Table SM17. Cost-utility analysis for total costs (societal perspective) with discount and disaggregated by sex and socioeconomic status.

	Base case		UPRIGHT (2 years)		Δ Cost	Δ QALY	ICUR
	**Cost**	**QALY**	**Cost**	**QALY**			
**Total**	12,757	10.81	12,643	10.82	-113.49	0.02	Dominant
**Low SES male**	28,256	10.72	27,933	10.73	-323.07	0.01	Dominant
**High SES male**	13,337	10.87	13,269	10.88	-67.51	0.01	Dominant
**Low SES female**	18,402	10.66	18,049	10.69	-353.39	0.02	Dominant
**High SES female**	10,467	10.77	10,347	10.79	-120.08	0.02	Dominant
	**Base case**		**UPRIGHT (5 years)**		**Δ Cost**	**Δ QALY**	**ICUR**
	**Cost**	**QALY**	**Cost**	**QALY**			
**Total**	12,757	10.81	12,558	10.83	-198.49	0.02	Dominant
**Low SES male**	28,256	10.72	27,798	10.74	-458.11	0.02	Dominant
**High SES**	13,337	10.87	13,205	10.88	-131.58	0.01	Dominant
**Low SES female**	18,402	10.66	17,857	10.69	-545.64	0.03	Dominant
**High SES female**	10,467	10.77	10,255	10.79	-211.97	0.03	Dominant
	**Base case**		**Systematic Review[38]**		**Δ Cost**	**Δ QALY**	**ICUR**
	**Cost**	**QALY**	**Cost**	**QALY**			
**Total**	12,757	10.81	12,722.80	10.82	-34.00	0.01	Dominant
**Low SES male**	28,256	10.72	28,102.83	10.73	-152.90	0.01	Dominant
**High SES male**	13,337	10.87	13,351.58	10.88	14.95	0.01	2,606.54
**Low SES female**	18,402	10.66	18,183.36	10.68	-218.92	0.02	Dominant
**High SES female**	10,467	10.77	10,412.02	10.79	-55.36	0.02	Dominant

QALY: quality-adjusted life year; ICUR: incremental cost-utility ratio.

Table SM18. Cost-utility analysis for total costs (societal perspective) without discount and disaggregated by sex and socioeconomic status.

	Base case		UPRIGHT (2 years)		Δ Cost	Δ QALY	ICUR
	**Cost (€)**	**QALY**	**Cost**	**QALY**			
**Total**	20,805	16.24	20,546	16.27	-258.18	0.03	Dominant
**Low SES male**	47,320	16.12	46,730	16.14	-589.86	0.02	Dominant
**High SES male**	21,529	16.36	21,348	16.37	-180.84	0.01	Dominant
**Low SES female**	30,620	16.00	29,972	16.04	-648.29	0.04	Dominant
**High SES female**	17,128	16.17	16,856	16.21	-272.07	0.04	Dominant
	**Base case**		**UPRIGHT (5 years)**		**Δ Cost**	**Δ QALY**	**ICUR**
	**Cost**	**QALY**	**Cost**	**QALY**			
**Total**	20,805	16.24	20,408	16.28	-396.79	0.03	Dominant
**Low SES male**	47,320	16.12	46,513	16.14	-806.88	0.02	Dominant
**High SES male**	21,529	16.36	21,244	16.37	-285.17	0.02	Dominant
**Low SES female**	30,620	16.00	29,660	16.06	-959.94	0.05	Dominant
**High SES female**	17,128	16.17	16,705	16.21	-422.49	0.05	Dominant
	**Base case**		**Systematic Review[38]**		**Δ Cost**	**Δ QALY**	**ICUR**
	**Cost**	**QALY**	**Cost**	**QALY**			
**Total**	20,805	16.24	20,668.78	16.27	-136.26	0.02	Dominant
**Low SES male**	47,320	16.12	46,989.03	16.13	-331.52	0.01	Dominant
**High SES male**	21,529	16.36	21,475.89	16.37	-53.50	0.01	Dominant
**Low SES female**	30,620	16.00	30,176.11	16.03	-444.33	0.03	Dominant
**High SES female**	17,128	16.17	16,955.21	16.20	-173.24	0.03	Dominant

Figure SM1. The resilience framework

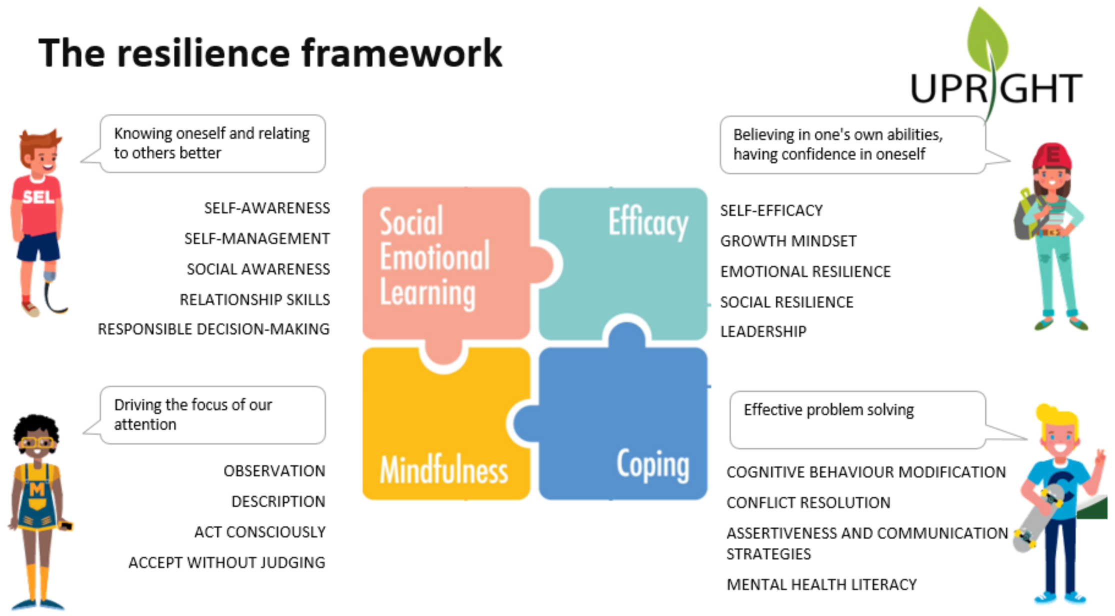

Figure SM2. Flow diagram of the discrete event simulation model.

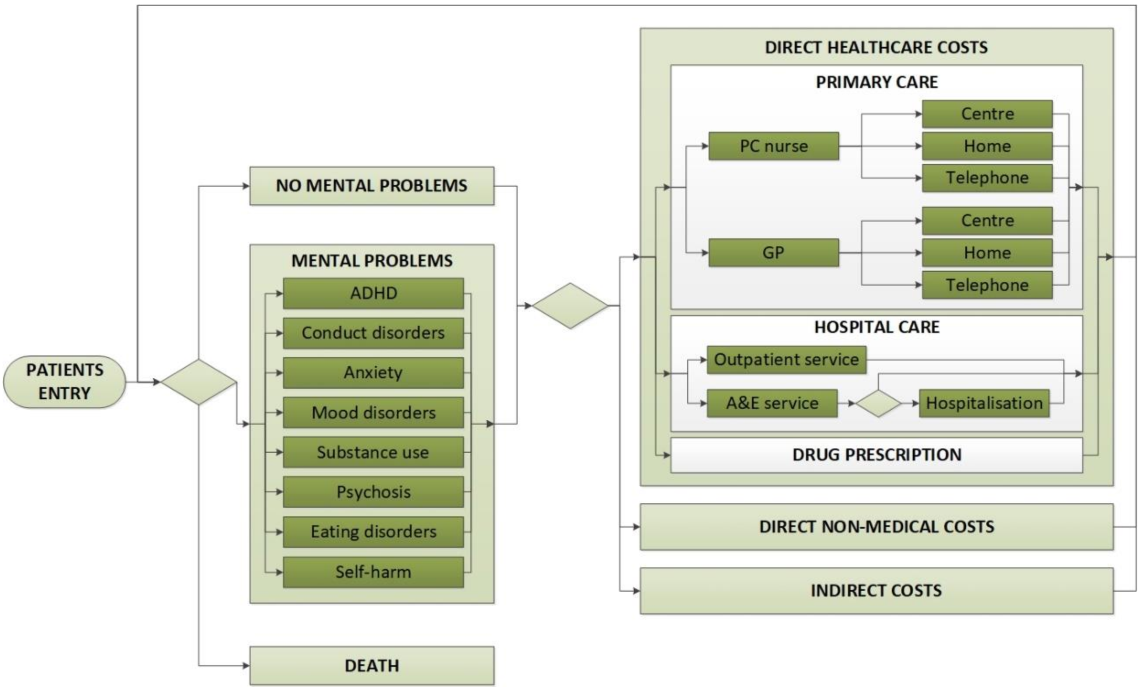

Supplement: Supplementary file 1 — Additional file 1. [file 12888_2023_4665_MOESM1_ESM.pdf]
